# Optimizing the structure of interdisciplinary tumor boards for effective cancer care

**DOI:** 10.3389/fonc.2023.1072652

**Published:** 2023-04-26

**Authors:** Friederike Braulke, Kathrin Kober, Andreas Arndt, Maximilian Papendick, Arne Strauss, Christof Maria Kramm, Kai-Martin Thoms, Alexander König, Jochen Gaedcke, Julia Gallwas, Svenja Wulf, Christoph Szuszies, Gerald Wulf, Ralph Rödel, Susanne Wolfer, Vesna Malinova, Tobias R. Overbeck, Marc Hinterthaner, Joachim Lotz, Friedemann Nauck, Marielle Ernst, Christine Stadelmann, Philipp Ströbel, Volker Ellenrieder, Thomas Asendorf, Stefan Rieken

**Affiliations:** ^1^ Comprehensive Cancer Center, University Medical Center Göttingen, Göttingen, Germany; ^2^ Department of Urology, University Medical Center Göttingen, Göttingen, Germany; ^3^ Division of Pediatric Hematology and Oncology, Department of Pediatrics and Adolescent Medicine, University Medical Center Göttingen, Göttingen, Germany; ^4^ Department of Dermatology, Venereology and Allergology, University Medical Center Göttingen, Göttingen, Germany; ^5^ Department of Gastroenterology, Gastrointestinal Oncology and Endocrinology, University Medical Center Göttingen, Göttingen, Germany; ^6^ Department of General, Visceral and Pediatric Surgery, University Medical Center Göttingen, Göttingen, Germany; ^7^ Department of Gynaecology and Obstetrics, University Medical Center Göttingen, Göttingen, Germany; ^8^ Department of Hematology and Medical Oncology, University Medical Center Göttingen, Göttingen, Germany; ^9^ Department of Otorhinolaryngology, University Medical Center Göttingen, Göttingen, Germany; ^10^ Department of Oral and Maxillofacial Surgery, University Medical Center Göttingen, Göttingen, Germany; ^11^ Department of Neurosurgery, University Medical Center Göttingen, Göttingen, Germany; ^12^ Department of Thoracic and Cardiovascular Surgery, University Medical Center Göttingen, Göttingen, Germany; ^13^ Institute for Diagnostic and Interventional Radiology, University Medical Center Göttingen, Göttingen, Germany; ^14^ Department of Palliative Medicine, University Medical Center Göttingen, Göttingen, Germany; ^15^ Department of Diagnostic and Interventional Neuroradiology, University Medical Center Göttingen, Göttingen, Germany; ^16^ Institute of Neuropathology, University Medical Center Göttingen, Göttingen, Germany; ^17^ Institute of Pathology, University Medical Center Göttingen, Göttingen, Germany; ^18^ Department of Medical Statistics, University Medical Center Göttingen, Göttingen, Germany; ^19^ Department of Radiotherapy and Radiation Oncology, University Medical Center Göttingen, Göttingen, Germany

**Keywords:** multi-professional tumor boards, radiology, pathology, specialized palliative care, radio-oncology

## Abstract

**Introduction:**

Multi-professional interdisciplinary tumor boards (ITB) are essential institutions to discuss all newly diagnosed, relapsed or complex cancer patients in a team of specialists to find an optimal cancer care plan for each individual patient with regard to national and international clinical practice guidelines, patient´s preference and comorbidities. In a high-volume cancer center, entity-specific ITBs take place at least once a week discussing a large number of patients. To a high level of expertise and dedication, this also requires an enormous amount of time for physicians, cancer specialists and administrative support colleagues, especially for radiologists, pathologists, medical oncologists and radiation oncologists, who must attend all cancer-specific boards according to certification requirements.

**Methods:**

In this 15-month prospective German single-center analysis, we examined the established structures of 12 different cancer-specific ITBs at the certified Oncology Center and demonstrate tools helping to optimize processes before, during and after the boards for optimal, time-saving procedures.

**Results:**

By changing pathways, introducing revised registration protocols and new digital supports we could show that the workload of preparation by radiologists and pathologists could be reduced significantly by 22.9% (p=<0.0001) and 52.7% (p=<0.0001), respectively. Furthermore, two questions were added to all registration forms about the patient´s need for specialized palliative care support that should lead to more awareness and early integration of specialized help.

**Discussion:**

There are several ways to reduce the workload of all ITB team members while maintaining high quality recommendations and adherence to national and international guidelines.

## Introduction

In the context of multi-professional cancer care in a comprehensive cancer center (CCC), interdisciplinary tumor boards (ITBs) represent the central structure for discussion of at least all newly diagnosed, relapsed, or complex cancer patients among a team of specialists. Cancer therapy has become increasingly complex, requiring the expertise of various physicians (surgery, medical oncology, radiation oncology, radiology, pathology, and others) as well as other healthcare professionals [oncology nurses, psycho-oncology, nutritional counseling, specialized palliative care (SPC) teams, social support, etc.] to design an optimal cancer treatment plan for each patient in accordance with national and international clinical practice guidelines. Numerous studies in different countries and focusing on different types of cancer have already analyzed the effectiveness and benefits of ITBs: they have shown that ITBs can indeed improve diagnostic accuracy, adherence to evidence-based guidelines, treatment recommendations, functional outcomes, and survival (1–10). Quality criteria for optimal multidisciplinary team meetings, including technical and administrative support, clear procedures for introducing patients to ITBs, notification of all members and guests, and written reporting of the recommendations of the ITB, have been described previously (10–13). ITBs represent the central structural element of certified organ-specific tumor centers, and the discussion of all cancer patients in ITBs is a requirement of the certification program of the German Cancer Society (GCS, Deutsche Krebsgesellschaft) (14). The initial results of the WiZen study (“Effectiveness of Care in Certified Cancer Centers in Germany,” ClinicalTrials.gov: NCT04334239) showed better median survival for patients with pancreatic cancer treated in GCS-certified pancreatic cancer centers compared to those treated in non-certified hospitals (8.0 *versus* 4.4 months) (15). In a high-volume cancer center, entity-specific ITBs take place at least once per week, with many cancer specialists and administrative support colleagues spending many hours preparing, attending, and documenting ITBs for better patient care.

At the University Medical Center Göttingen, Germany, there is a structured workflow for all boards with defined timeslots, rooms, responsibilities, and chairpersons. ITBs take place in the form of physical meetings with the additional possibility of virtual participation. They are documented and managed using the tumor documentation system ONKOSTAR^®^ (IT-Choice Software AG, Karlsruhe, Germany). Every physician has personal access to the system and can register patients for ITBs. The registration forms include information about the cancer diagnosis, recent histopathological and radiological data, and the patient’s history and comorbidities. Educated oncology nurses, as well as breast and cancer care nurses, are always welcome to attend ITBs, and may do so depending on their availability or if requested. All ITB members and presenting physicians will be informed of the agenda by the cancer center *via* email. The cancer center is responsible for the management and administrative support of all ITBs. Administrative ITB coordinators for each board and each organ-specific tumor center are educated members of staff for medical documentation at the CCC. They prepare the respective ITBs in terms of checking the completeness of registrations, checking for imaging availability, and inviting additional specialists to the ITB if required by the responsible physician. During the ITB, they register the attendance of all members and guests and document the recommendations of the ITB team. Altogether, 12 ITB coordinators including staff for medical documentation, one technical assistant and one secretary for organization are involved in the CCC management of all ITBs. The announcement and documentation of recommendations, as well as all imaging procedures presented by radiologists, are shown on screens visible to all members attending the meeting. Videoconferencing for external partners is also possible. All disciplines needed for decision-making are represented in the ITB, and most stakeholders already work together in multi-professional certified organ-specific tumor center teams within the certification program. Palliative care specialists are not routinely present at all ITBs, but attend according to their availability or if requested; they are involved in inpatient and outpatient care through fully established counseling services. After the ITB, recommendations are recorded and finalized by ITB coordinators, approved by the responsible panel chair, forwarded to all participants in the ITB *via* ONKOSTAR^®^, and stored in individual electronic patient files. All cancer patients have access to the ITBs at the CCC. Standard operating procedures (SOPs) summarize the indications, responsibilities, and workflow of ITB presentations for the CCC. Over a period of observation, 97% of all cancer patients (range: 91%–100%) were discussed in ITBs according to the criteria of the German Cancer Society (14, 16).

Time and workload pressures exert a negative influence on decision-making in ITBs (17). A well-known problem with ITBs are time-consuming discussions due to missing information e.g. on patient´s comorbidities and wishes, tumor stage, imaging, pathology, prior therapies or examinations, which are necessary for reliable and sustainable decision-making (18). Other time-consuming procedures for administrative ITB coordinators at the cancer center include the preparation of incomplete registration forms before the ITB and the documentation of handwritten or dictated protocols afterward. In addition, radiologists and pathologists usually prepare not only the latest specimens but also all images and tissues available for the patients discussed in ITBs because of imprecise questions, or a lack of questions, prior to the ITB. All ITBs are scheduled for 1 h and include up to 20 patients for discussion, but as the number of patients increases, the duration of the meeting and the number of cases discussed are routinely extended.

Due to continuous increases in the number of patients discussed at ITB meetings on the one hand and increasing time pressure and the limited resources of medical experts on the other hand, there is a need for optimization of established procedures. The aim of this study was to identify various options for optimization of ITB structures. In a systematic single-center prospective analysis over 15 months, we scrutinized the established structures of 12 different cancer-specific ITBs at the certified Oncology Center of the Comprehensive Cancer Center at University Medical Center Göttingen, Germany, to optimize the processes carried out before, during, and after the ITBs for optimal and time-saving procedures and continued high quality according to national and international guidelines. We definded the required consequences, introduced new tools and approaches to all ITBs, and evaluated their effectiveness. Here, we present the final results.

## Materials and methods

In a four-phase systematic process over 15 months (**Table 1** and **Supplementary Material Table 1**), established ITB procedures were analyzed (phase 1: November 2020–January 2021), a series of new approaches and tools were defined (phase 2: February 2021) and implemented sequentially in all ITBs (phase 3: March 2021–November 2021), and an evaluation of effectiveness was conducted (phase 4: December 2021–January 2022). Between March 2021 and January 2022, a multi-professional working group for optimization of ITB structures (FB, KK, SR) reviewed all processes and structures, as well as the duration and workload, of 12 cancer-specific ITBs of organ-specific tumor centers (certified according to the criteria of the German Cancer Society for gastrointestinal (GI) cancer, skin cancer, pediatric cancer, urological cancer, breast cancer, gynecological cancer, hereditary cancer, sarcoma and rare cancer, hematological diseases, head and neck cancer, lung cancer, and cancer of the central nervous system) at the certified Oncology Center of the Comprehensive Cancer Center at University Medical Center Göttingen, Germany. The review was conducted *via* a structured survey of all ITB chairs and organ center teams; the major question was “What are the most time-consuming steps during registration, preparation, implementation, discussion, documentation, and finalization of ITB recommendations?”

**Table 1:** Innovations and changes to ITB processes between March 2021 and January 2022.

Administrative changes	Revision of all digital registration forms
	Addition of more required fields
	Board-specific questions on imaging and investigations
	Additional question to the responsible physician: “Is the detailed preparation of all images by radiologists really necessary to answer the question to the board?”
	Is the attendance of a palliative care specialist necessary in cases of impaired performance status (ECOG ≥ 3, Karnofsky ≤ 50%)?
	"Additional question to the responsible physician: Is the detailed preparation of all tissues by pathologists really necessary to answer the question to the board?"
	Additional field: “interdisciplinary discussion prior to ITB”
Technical changes	Introduction of a digital voice recognition system
	Provision of technical equipment in two additional conference rooms serving six ITBs

The observation period for evaluation was from March 2021 until January 2022. A systematic survey for the purpose of workload assessment was performed at three time points (pre-intervention in March 2021; interim analysis in October 2021; and final analysis in January 2022) by counting the number of cases prepared by radiologists and pathologists and recording the self-reported workload of ITB coordinators before and after the board. The duration of each board, from the beginning to the end of the meeting, was documented by ITB coordinators at the onset of the implementation of new tools in March 2021 and then continuously for each meeting from September 2021 until January 2022 (**Table 2**). The median duration of ITB discussion per case was calculated by dividing the duration time of each ITB by the number of cases discussed. Different levels of complexity of cases were not considered. Multiple changes were implemented simultaneously or in quick succession (**Table 1** and **Supplementary Material Table 1**); thus, no evaluation of the impact of any single new tool in contrast to another (head-to-head comparison) was planned.

**Table 2:** Characteristics of 12 distinct tumor boards.

	March 2021	September 2021	October 2021	November 2021	December 2021	January 2022
Number of cases discussed	657	687	629	692	633	741
Mean (SD) time of discussion/case (min)	4:05 (1:56)	3:42 (1:36)	3:35 (1:32)	3:27 (1:09)	3:31 (1:32)	4:09 (2:09)
Median (range) time of discussion/case (min)	3:47 (1:15-10:00)	3:08 (2:05-10:00)	3:23 (1:40-7:30)	3:20 (1:54-6:40)	2:51 (1:33-7:30)	3:53 (1:32-12:30)
Mean (SD) meeting duration across all ITBs (min)	51:35 (32:46)	44:56 (24:34)	44:48 (24:31)	47:51 (25:00)	47:35 (19:19)	53:23 (28:19)
Median (range) meeting duration across all ITBs (min)	42:30 (15:00-150:00)	45:00 (5:00-110:00)	42:00 (5:00-125:00)	45:00 (7:00-120:00)	45:00 (15:00-95:00)	50:00 (15:00-165:00)
Cases (proportion) prepared in detail by radiology	635 (100%)	492 (71.4%)	479 (76.2%)	552 (78.9%)	467 (74.1%)	547 (73.9%)
Cases (proportion) prepared in detail by pathology	635 (100%)	322 (46.8%)	293 (46.8%)	350 (49.9%)	302 (47.9%)	359 (48.3%)

In total, between March 2021 and January 2022, 7,123 cases were discussed.

Descriptive statistics on the number of cases and efficiency per case were calculated [*n*, mean, standard deviation (SD), median, range] and visualized through local polynomial regression fitting (LOESS) with 95% pointwise confidence intervals. Changes in the number of cases, efficiency of case handling, and the proportion of cases prepared by pathology and radiology staff were assessed using mixed linear models with a random intercept by corresponding ITB. Changes induced by the use of digital voice recognition, differences in preparation time for radiologists and pathologists, the use of a new registration form, and interdisciplinary discussion, as well as improvements to the technical equipment provided in the conference rooms, were assessed using estimated marginal means and are reported with 95% confidence intervals (19). Time was considered as a covariate to account for the possibility of underlying linear time trends in this before–after design. Analysis was performed using R version 4.2.0 (20).

## Results

Between November 2020 and January 2021, all ITB processes were analyzed. The following challenges relating to preexisting ITB procedures were identified:

1) For ITB coordinators: time required for preparation of all registrations to the board, and documentation and completion of recommendations in the tumor documentation system during and after the ITB;
2) For radiologists and pathologists: burden of time and workload for detailed preparation of all images and specimens from the patient’s history, despite these not being subsequently needed for ITB discussion in many cases; and
3) For physicians during the ITB: missing relevant information due to incomplete preparation and provision.

Based on these findings, the measures necessary to overcome these challenges were defined by a multi-professional working group together with the ITB chairs (**Table 1**). The following changes to ITB processes were implemented consecutively between March 2021 and January 2022:

1) In conjunction with the ITB chairs, the patient registration form for each ITB was revised to include more required fields and to collect more mandatory data and information *via* individual board-specific questions on entity-specific imaging [e.g., positron emission tomography (PET)/computed tomography (CT) or endoscopic retrograde cholangiopancreatography (ERCP)] and investigations (e.g., hormone receptor status, tumor markers, blood counts) for integral discussions. The new registration form was introduced immediately after the preceding ITB; thus, all new registrations for the next week were recorded on the new forms without overlap of two different registration forms in the washout phase.
2) For all ITBs, questions were added as to whether the detailed preparation of all imaging examinations by radiologists or all tissues and specimens by pathologists would really be necessary to answer the questions posed during the board.
3) For urgent cases, which were spontaneously discussed by a multidisciplinary team of senior physicians (e.g., surgeon, radiation oncologist, and medical oncologist) in the emergency room or at another conference and presented at the ITB some days later, a special label was introduced (“interdisciplinary discussion prior to ITB”) to document any prior treatment decision again as part of the subsequent ITB registration.
4) From May 2021, a digital voice recognition system (Dragon Medical, WMC Diktiersysteme GmbH, Dortmund, Germany) was introduced sequentially to all ITBs to transcribe the board’s recommendations with live visibility for all participants. The ITB for lung cancer had already used this system since 2018, and the other boards (for breast and gynecological cancer, urological cancer, hematological diseases, sarcoma and rare diseases, pediatric cancer, skin cancer, and head and neck cancer) now also did so until the end of the observation period for this analysis (**Table 1** and **Supplementary Material Table 1**). Three months after the end of the observation period, the ITBs for neuro-oncology and GI cancer also decided to introduce the digital voice recognition system. Repeated individual and team training sessions for all physicians using this speech recognition system were provided by the cancer center’s information technology (IT) specialists. During this technical adjustment phase, additional IT assistants were present at every ITB to help in case of technical or user problems with the new digital tool.
5) Two additional ITB conference rooms were technically equipped with new workstations and computers for radiologists and administrative ITB coordinators, at least two large flat screens with high-resolution projectors for the presentation of radiological images and patient history in the tumor documentation system at the same time, a videoconferencing tool to enable external physicians to attend the ITB, a telephone system, and the abovementioned digital voice recognition system. Thus, six ITB teams underwent changes to their conference room during the observation period.

In revising all registration forms for the ITBs, we took the opportunity to add two further questions to all registration forms concerning the need for SPC support: first, in cases of impaired performance status (ECOG ≥ 3, Karnofsky ≤ 50%), the physician was asked whether the attendance of a palliative care specialist would be necessary; and second, at the end of the ITB registration form, the physician was asked whether SPC support was required or had already been commissioned.

**Figure 1:**
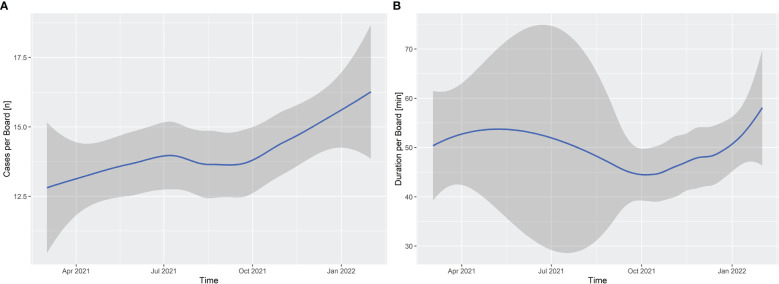
Interdisciplinary tumor boards: **(A)** the mean number of cases discussed per board meeting across 12 ITBs (total *n* = 7,123) between March 2021 and January 2022; **(B)** the median duration of interdisciplinary tumor board meetings (min). The gray area represents pointwise 95% confidence intervals.

Between March 2021 and January 2022, the number of cases discussed (*n* = 7,123) increased steadily across all ITBs (**Figure 1A**). The mean duration of discussion per patient across all ITBs decreased from 4:05 min (SD = 1:56; median = 3:47; range = 1:15–10:00) in March 2021 to 3:35 min (SD = 1:32; median = 3:23; range = 1:40–7:30) in October 2021, but it increased again in January 2022 to a mean of 4:09 min (SD = 2:09; median = 3:53; range = 1:32–12:30) due to technical problems with the digital voice recognition system and videoconferencing. We found that the digital voice recognition system should not be switched between different conference rooms due to profile recognition. It is a self-learning program that needs to be trained by the physician who records the ITB recommendations because several drug names and names of clinical trials are unknown to the system. At the onset of implementation, these technical challenges for physicians relating to the new speech recognition system, as well as user and technical problems with the internet connection for videoconferencing, resulted in an increased duration time for ITBs. The average duration time per meeting across all ITBs was initially reduced from 51:35 min (SD = 32:46; median = 42:30; range = 15:00–150:00) in March 2021 to 44:48 min (SD = 24:31; median = 42:00; range = 5:00–125:00) in October 2021, but it increased back to 53:23 min (SD = 28:19; median = 50:00; range = 15:00–165:00) in January 2022, again because of technical challenges (**Figure 1B**).

**Figure 2:**
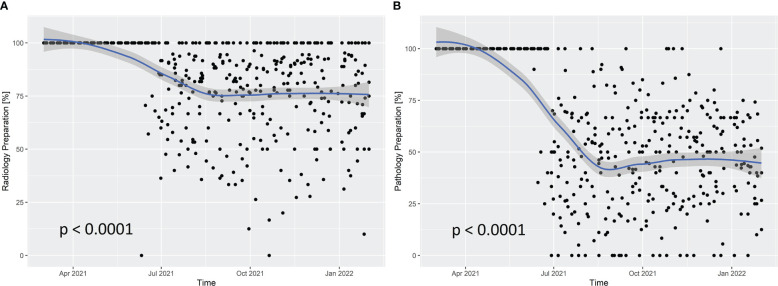
Cases prepared in detail for presentation at interdisciplinary tumor boards by radiologists **(A)** and pathologists **(B)** between March 2021 and January 2022. The gray area represents pointwise 95% confidence intervals.

The new individual ITB registration forms for each ITB, with improved preparation of announced cases and the new specific question on whether the radiologist needed to prepare all available images of the patient in order to answer the question to the board, reduced the preparation workload for radiologists significantly by 22.9% (95% CI: 18.6%-27.3%; *p* ≤ 0.0001; **Figure 2A**). In addition, the new specific question on whether the pathologist needed to prepare all patient’s tissue samples and make them available in detail in order to answer the question to the board significantly reduced the preparation efforts for pathologists by 52.7% (95% CI: 47.1%-58.2%; *p* ≤ 0.0001; **Figure 2B**).

The mean amounts of preparation time before and after the ITB for administrative ITB coordinators in March 2021 and in September to December 2022 were 66.0 and 46.5 h/week, respectively; thus, this preparation time was reduced by 29.5%.

The occurrence of an interdisciplinary discussion prior to the ITB was documented in 10.6% (62/585) and 6.9% (43/626) of cases discussed in the ITB in October 2021 and January 2022, respectively, mainly by the ITBs for GI cancer, central nervous system, and hematological diseases.

As a result of the introduction of the new registration forms, the discussion time per patient decreased on average from 4.79 min/case (95% CI: 3.39-6.18) to 4.17 min/case (95% CI: 3.12-5.22; *p* = 0.2349) across all ITBs. With the launch of the digital voice recognition system, the discussion time per patient increased on average from 4.31 min/case (95% CI: 2.88-5.74) to 4.65 min/case (95% CI: 3.27-6.03; *p* = 0.2312) across all ITBs. The other factors examined (i.e., room change, the proportion of cases subjected to interdisciplinary discussion before the ITB, and the proportion of cases prepared by pathology or radiology) showed no significant influence on the efficiency of case discussions.

In October 2021, impaired performance status (ECOG ≥3 or Karnofsky <50%) was documented for 2.9% of patients (17/585), and SPC support was requested for 8/17 patients (47.1%). In January 2022 impaired performance status was documented for 4.2% of patients (26/626), with SPC support requested in 30.7% of cases (8/26). At the same time, a further question was introduced to the ITB registration forms: “Do you think that your patient is in need of SPC support?” In October 2021, a need for SPC support was claimed in relation to 9% of patients (46/509) across all the ITBs; for 350 patients (68.8%), no need for SPC support was declared; one patient (0.2%) had rejected palliative care support; in the cases of 10 further patients (1.9%), palliative care specialists had already become involved in cancer care; and for 102/509 patients (20%), there were no data available. In January 2022, a need for palliative care was claimed for 9.4% of patients (52/555); for 370 further patients (66.7%), SPC was not necessary; three patients had rejected palliative care support; and in the cases of 14 patients (2.5%), SPC was already applied. In 116/555 further cases (20.9%), no data were available.

## Conclusions

Multi-professional discussions of all cancer patients in structured ITBs represent the standard of patient-centered cancer care today. Interdisciplinary team meetings and decision-making can improve diagnostic precision and adherence to guidelines, including changes in treatment recommendations (1–10). Adhering to the recommendations on quality criteria for ITBs (11) and ensuring compliance with the requirements of national certification programs to ensure high-quality recommendations and adherence to national and international guidelines (12, 16) are time-consuming processes for many physicians and other medical specialists. Our single-center analysis has shown that there are several steps before, during, and after ITB meetings that can be optimized, leading to more effective discussions during the board meeting and a decreased workload beforehand and afterward.

As expected, and due to increasing numbers of cancer diagnoses in general (21), our data show a continuously growing number of cancer patients that need to be discussed in ITBs. Checklists can help with preparation of the ITB discussion to enable better decision-making and improvements in quality (12, 22, 23). Our revised forms for ITB patient registration serve as a checklist for each physician to prepare all the information needed for optimal presentation during the ITB. During the observation period, the discussion time per case was approximately 3.5–4 min, which is in line with published data reporting discussion times of approximately 4 min per patient (24, 25). Lamb and colleagues (22) described a mean discussion time per patient of 3 min, but mentioned that other factors may influence individual discussion time, such as the complexity of a given case, completeness of the presented information, and digital tools such as videoconferencing (22).

Radiologists and pathologists are always present at all ITBs; for this reason, the related workload is particularly high for them. The written results of all recent radiological and pathological findings must be available at the time of the ITB presentation. Prior to the introduction of the changes, radiologists and pathologists usually re-prepared these recent images or specimens in detail and in the context of all the images and specimens from the patient’s history for ITB presentation, because the registration form and the question to the board were often unclear. As a result of the introduction of the new registration forms, the process of preparation for case discussion was improved by the submission of a mandatory clear question to the board; thus, the responsible physician, as well as the radiologist and pathologist, could decide whether such a detailed presentation of all pathological or radiological results would be necessary to answer the question to the board. We were able to demonstrate that radiologists’ and pathologists’ workloads in terms of preparation before the ITB could be significantly reduced by improved registration procedures. During the brief observation time, neither the new registration forms nor the digital voice recognition system showed a significant impact on discussion time per patient during the board, but both reduced the workload for ITB coordinators before and after the ITB, as the board members were fully aware of the patients who had been registered and who were to be discussed, and the complete set of recommendations were visible to all ITB members during the board meeting.

The new option to document treatment decisions that had been made prior to the ITB by a multi-professional team of senior physicians, in cases of emergency (where urgent decision-making is necessary) or prior non-ITB case discussions among different experts (e.g., a stem cell transplantation unit), could help to increase the quality of documentation and discussion during the ITB. This option was used by physicians in relation to a stable and reasonable percentage of patients (6.9%–10.6%) during the observation period.

The integration of specialized palliative care into cancer care plans is a designated aim for optimal patient care (26–28); this care can be provided by, e.g., independent palliative tumor boards, specialized programs, or boards and teams to which patients can be referred (26, 28–30). Instead of implementing an additional tumor board for palliative care, we chose to take the opportunity to include more palliative care specialists in our existing ITBs: through the addition of two short questions about the potential need for SPC support to the patient registration form for each ITB, by way of an objective screening, the responsible physician was required to reflect on the patient’s needs. These two additional questions were accepted by all participating physicians and were addressed responsibly and carefully; thus, their inclusion might lead to greater awareness of the possibility of early integration of palliative care. The incorporation of SPC considerations into regular ITB discussions was not the reason for the prolonged discussion time, which was attributable to technical challenges with new digital tools. The duration of ITBs increased again a few months after the conversion to digital voice recognition because of technical problems during ITBs, such as user problems, missing profile recognition, or misunderstandings by the software, especially of the names of drugs, antibodies, or clinical trials. At the beginning of the technical rollout, IT assistants were also present at the ITBs to help physicians with the new digital tool. After some time, only ITB coordinators were present, who were not always able to handle all technical challenges; therefore, IT support continues to be made available for all ITBs. Our data show that a different support staff profile might be needed in ITBs when meetings undergo a switch to more digitalized workflows with the introduction of digital voice recognition systems and videoconferencing. Administrative ITB coordinators benefited from the new registration forms with more required fields and clear questions to the board, because they did not need to manually collect all the data necessary for ITB discussion prior to the board. The presenting physician was responsible for providing all relevant data for optimal discussion during the ITB. Transcription of the ITB recommendations during the board meeting, in a form visible to all ITB members, avoided the need for ITB coordinators to type out the recommendations afterward. In line with the literature, our data show that digital tools can contribute to the optimization of ITB structures (24, 25, 31). In turn, more IT-specialized or IT-trained staff may be needed to assist cancer experts during the boards.

A limitation of this analysis is the single-center setting. For future studies, structured multicenter approaches should be preferred.

To summarize, our data show that there are several tools that can help to reduce the workload of all ITB team members while maintaining the provision of high-quality recommendations and adherence to national and international guidelines. We were able to show that it is possible to make significant reductions, e.g., to the preparation time of pathologists and radiologists prior to the board through the use of new registration forms with improved announcement of cases and clear designation of the question to the board, without disregarding the quality requirements for certification programs. Nevertheless, ITBs are still time-consuming, but they are necessary for optimal cancer patient care. A combination of IT solutions together with repeated training for all participants to improve case preparation and discussions, as well as technical and administrative support, can help to improve efficacy and turnaround time in order to keep pace with increasing case numbers.

## Data availability statement

The original contributions presented in the study are included in the article/**Supplementary Material**. Further inquiries can be directed to the corresponding author.

## Author contributions

Study conception and design: FB, KK, VE, TA, and SR. Collection and collation of data: all authors. Data analysis and interpretation: FB, KK, TA, and SR. Drafting or revision of the manuscript: FB, TA, and SR. All authors contributed to the article and approved the submitted version.
