# Supplementary material for: Optimizing the structure of interdisciplinary tumor boards for effective cancer care
Source: Front Oncol. 2023 Apr 26;13:1072652. doi: 10.3389/fonc.2023.1072652 (PMC10171921; doi:10.3389/fonc.2023.1072652)
Supplement: Supplementary file 1 [file DataSheet_1.pdf]

Table 1: Flowchart of the 4-phase systematic process (11/20 – 01/22)

	NOV 2020	DEC 2020	JAN 2021	FEB 2021	MAR 2021	APR 2021	MAY 2021	JUN 2021	JUL 2021	AUG 2021	SEP 2021	OCT 2021	NOV 2021	DEC 2021	JAN 2022
**Analysis of ITB structures**															
**Definition of consequences required**															
**Implementation of new tools in all ITBs**															
**Workload assessment**															
**Evaluation of effectiveness**															
**New registration form**															
hereditary cancer															
breast cancer															
gynecological cancer															
gastrointestinal cancer															
skin cancer															
pediatric cancer															
urological cancer															
sarcoma and rare cancer															
hematological diseases															
head and neck cancer															
lung cancer															
cancer of the central nervous system															
**Digital voice recognition system**															
hereditary cancer															
breast cancer															
and gynecological cancer															

gastrointestinal cancer															
skin cancer															
pediatric cancer															
urological cancer															
sarcoma and rare cancer															
hematological diseases															
head and neck cancer															
lung cancer															
cancer of the central nervous system															
**New (better equipped) room**															
hereditary cancer															
breast cancer															
gynecological cancer															
gastrointestinal cancer															
skin cancer															
pediatric cancer															
urological cancer															
sarcoma and rare cancer															
hematological diseases															
head and neck cancer															
lung cancer															
cancer of the central nervous system															
